# Illustrations of the Efficacy of Copious Blood-Letting in the Cure of Mania

**Published:** 1820-01

**Authors:** W. Hamilton

**Affiliations:** Surgeon.


FOR THE LONDON MEDICAL AND PHYSICAL JOURNAL.

### *Illustrations of the Efficacy of copious Blood-letting in the Cure of Mania.* By W. Hamilton, Esq. Surgeon.

IN the Number of your Journal for November last, I perused with much pleasure the history of a case of the successful treatment of mental derangement by a most active and continued bleeding and purging, &c. related by your able correspondent, Dr. Parkinson; and, leaving the merits of his new arrangement of diseases to the test of time, I beg you will allow me to trespass on your patience for a few minutes, while I add a few examples in confirmation of the principles and practice there pursued. This I conceive will be the more excusable at this time, from the increasing number of those afflicted with this melancholy disorder; arising, no doubt, in many instances, from the pressure of the times.

Wishing, therefore, to convey the substance of these cases in as small a compass as possible, I shall briefly give their leading features, leaving others to judge of the utility of the practice.

Mrs. Eathersy, ætat. 29, married, and has had three children, (the younger about a year and a half old,) began to complain, on the 10th of July, of much pain of the head and great agitation of mind. She was constantly walking about. In the course of a few days she became quite outrageous, fancying she was pursued by thieves and murderers. She was immediately bled about twenty ounces, and confined. Next day she was actively purged with jalap and calomel; after which, she was so much better as to require no farther medical attention than low diet, and an occasional repetition of the purge. About four months after, the husband having left her in great distress, she experienced a return of the complaint, when another profuse bleeding and a vomit removed the symptoms; since which she has been as well as usual.

Mrs. Hammond, ætat. 58, the mother of three children, of spare habit, generally supporting herself by washing and sewing, &c. had been complaining for several weeks of a dyspeptic disorder, which latterly affected her head; and she now began to show evident symptoms of mental derangement, so that she was obliged to be removed to the parish workhouse. Here, on the 2d of July, it was found absolutely necessary to confine her with the waistcoat. She was now bled about twentyfour ounces, her head ordered to be shaved, and kept constantly wetted with cold vinegar and water; diet, gruel, &c. 3d, continued much the same. The 4th, being still more talkative and outrageous, was again bled to the amount of twenty ounces, and a dozen leeches applied to the temples in the evening. The bowels were kept open with calomel and jalap; and the diet very low. She now remained somewhat quieter for the two following days, but without any amendment of her mental faculties. On the 8th, she was again bled near thirty ounces, or till she fainted; after which, she became so low for several days, that she was allowed a glass of negus twice a-day, with some soup, &c. From this time she began to give rational answers, and seldom talked incoherently; so that in a few days more, on the 14th, she was able to be trusted without the waistcoat, although unable to sit up. By the continuance of a spare but nutritious diet, with the occasional use of a purgative medicine, she continued to recover her mental faculties, as well as bodily health; and by the end of the month required no farther medical attention, till her death, which occurred a few weeks ago, from mortification of the lower extremities.

James B—, ætat. 36, a tall athletic man, of dark complexion, by trade a shipwright, and now out of employ, began, about the 1st of November, to show evident symptoms of a return of the complaint, with which he had been affected for near six months, about five years ago. He was now all gloom and melancholy, and bent on self-destruction; harassed by religious phantoms; and seeking every opportunity of eluding the vigilance of his keeper. In this state, with a pulse nearly natural, and no apparent bodily indisposition, he was bled, on the 6th, to the quantity of twenty-four ounces, which produced little effect; had his hair removed, and head kept constantly wetted; drank plentifully of sulphat of magnesia with manna, so as to keep the bowels open, &c. On the next day twelve leeches were applied to the temples, as there was no abatement of the symptoms. 8th, continued much the same, except rather more violent, requiring to be quite tied down. Twenty ounces of blood were taken from the jugular vein on the 9th; and, on the 10th, sixteen more leeches were applied to the temples, and a perpetual blister to the neck with savine unguent, which discharged well. He had also pills of calomel and scammony twice a-day; so that a constant irritation was kept on the bowels, producing three or four evacuations daily. Notwithstanding this, and the most spare diet, (being only tea and gruel,) there appeared little or no abatement of the mental disorder, and the pulse was now generally about 100. This determined me, on the 12th, to take away a still larger quantity of blood from the head, as the seat of the complaint still laid there, and his strength appeared but little reduced by the former bleedings. About twenty-four ounces were therefore taken from the neck, when syncope ensued, and he continued very low the remainder of the day; and, having taken twenty grains of James's powder at bed-time, obtained some sleep in the night, for the first time since the attack. On the 13th he was very low, but gave rational answers, and was much more composed: was now allowed some wine and water; and tartarized mercurial unguent rubbed well in all over his head, and inside the thighs, twice a-day. 16th, continues composed; sits up several hours a-day; and now complains much of the effects of the mercury on the gums, which is omitted; the blister on the neck still discharging well. —18th. From this period, he required no farther medical attention than the change of air in the country and a restricted diet; is quite composed, and takes gentle exercise.

I might mention another instance or two of the success of this treatment; in one of which the mental affection succeeded the small-pox, and, although obstinate, the subject of it has enjoyed good health for some years since.

My object in relating the above cases is not the vain idea of offering any thing new on the subject, but to show that we are not to look so much to the quantity of blood withdrawn from the patient as the effect produced on the constitution in the commencement of cases of this description; as there can be no doubt of the nature and seat of the disease, which, by running its uncontrolled course, will produce either organic lesion or serous effusion in the head, entailing a continuance of the disorder, without our having another opportunity of remedying it.

*Ipswich; November* 1819.

